# Manipulation of Viral MicroRNAs as a Potential Antiviral Strategy for the Treatment of Cytomegalovirus Infection

**DOI:** 10.3390/v9050118

**Published:** 2017-05-19

**Authors:** Jiang Deng, Jun Xiao, Ping Ma, Bo Gao, Feng Gong, Liping Lv, Yanyu Zhang, Jinbo Xu

**Affiliations:** 1 Beijing Key Laboratory of Blood Safety and Supply Technologies, Beijing 100850, China; ammsdjxm@163.com (J.D.); maping1111@hotmail.com (P.M.); gaobolove@sina.com (B.G.); gongfeng@nic.bmi.ac.cn (F.G.); 2 Beijing Institute of Transfusion Medicine, 27 (9) Taiping Road, Beijing 100850, China; 3 Department of Blood Transfusion, Air Force General Hospital, Beijing 100142, China; ammsxj@126.com

**Keywords:** cytomegalovirus, viral miRNA, antiviral therapy, transfection

## Abstract

Cytomegalovirus (CMV) infection leads to notable morbidity and mortality in immunosuppressed patients. Current antiviral drugs are effective but seriously limited in their long-term use due to their relatively high toxicity. In the present study, we characterized the expression of murine CMV microRNAs (MCMV miRNAs) both in vitro and in vivo. Although 29 miRNAs were detectable during in vitro infection, only 11 miRNAs (classified as Group 1) were detectable during in vivo infection, and as many as 18 viral miRNAs (classified as Group 2) were less detectable (<50% of animals) in both the liver and lungs. In addition, viral miRNA profiles in the blood revealed unstable and reduced expression. We next explored the in vitro effects of viral miRNAs on MCMV replication. The inhibition of Group 1 viral miRNAs had little effect on virus production, but transfected cells overexpressing miR-m01-3-5p, miR-M23-1-5p, miR-M55-1, and miR-m107-1-5p in Group 2 showed statistically lower viral loads than those transfected with control miRNA (29%, 29%, 39%, and 43%, respectively, versus control). Finally, we performed hydrodynamic injection of viral miRNA agomirs and observed lower levels of MCMV recurrence in the livers of animals overexpressing the miR-m01-3-5p or mcmv-miR-M23-1-5p agomirs compared with those of animals transfected with control agomir, confirming the antiviral effects of viral miRNA manipulation in vivo. Therefore, the manipulation of viral miRNA expression shows great therapeutic potential and represents a novel antiviral strategy for the miRNA-based treatment of cytomegalovirus infection.

## 1. Introduction

Human cytomegalovirus (HCMV) is a member of the *Herpesviridae* family, with a high prevalence greater than 50% [1]. Primary infection is usually self-limiting, appearing to be asymptomatic inimmunocompetent individuals. However, HCMV infection is of particular concern when host defenses are compromised, leading to increased morbidity and mortality [2]. Current drugs (e.g., ganciclovir and foscarnet) successfully inhibit cytomegalovirus (CMV) infection. However, the use of these drugs is seriously limited in clinical practice due to an increased risk of adverse effects [3,4]. In addition, the emergence of drug-resistant strains of CMV following the repeated use of these drugs has been reported in detail [5,6,7]. Therefore, new antiviral therapies are needed to prevent CMV infection in immunodeficient patients. 

MicroRNAs (miRNAs) are short non-coding RNA molecules that regulate gene expression at the posttranscriptional level. To date, more than 230 viral miRNAs have been identified, the majority of which are encoded by herpesviruses [8]. The exact roles of viral miRNAs remain poorly characterized in many cases, although they are widely believed to participate in the mechanisms by which viruses manipulate the expression of both their own and the host genome during lytic or latent infection [9,10,11].

According to in vitro studies, CMV miRNAs play important roles in the regulation of viral replication [12,13,14,15,16,17,18,19], immune modulation [20,21], and immune evasion [22,23,24]. Recently, HCMV miR-UL22A-5p was identified as a potential biomarker for transplantation, suggesting that miRNAs encoded by HCMV are associated with specific virologic and clinical outcomes [25]. However, further investigation has been limited due to the rigorous species specificity of HCMV. Thus, mice infected with murine cytomegalovirus (MCMV) are used as a tool to study the biology of CMV infection in vivo [26,27].

In this study, we investigate and characterize the expression of MCMV miRNAs both in vitro and vivo. In vitro MCMV miRNA profiles differed from in vivo profiles, and some miRNAs were undetectable during MCMV replication in animals. Furthermore, several viral miRNAs that were rarely expressed in vivo played important roles in MCMV production—overexpression of these miRNAs led to impaired viral replication. Thus, the manipulation of viral miRNA expression is a promising potential therapy and represents a novel antiviral strategy. 

## 2. Materials and Methods

### 2.1. Cell Culture and Viral Titers

MCMV (Smith strain) was routinely inoculated and propagated in mouse embryonic fibroblast (MEF) cells maintained in Dulbecco’s modified Eagle medium (DMEM, Gibco, Shanghai, China) supplemented with 10% fetalbovine serum (FBS), and aliquots were stored at −80 °C.

Viral titers were assessed using a modified 50% tissue culture infective dose (TCID_50_) assay, as previously described [28]. Briefly, MEFs were cultured in a 96-well plate and inoculated with serial dilutions of MCMV or centrifuged supernatant from liver homogenates from infected mice. The cells were incubated for one week, and then assayed for the presence or absence of cytopathic effects, according to the method of Reed and Muench [29]. For viral titers from the liver, the limit of detection (LOD) was 45.85 plaque-forming units (PFU)/100 mg tissue.

### 2.2. Detection of MCMV MiRNAs In Vitro

Primers to detect MCMV miRNAs were designed using miRprimer software program (Version 2.0; https://sourceforge.net/projects/mirprimer), as reported previously [30]. At least three pairs of primers were initially designed for each MCMV miRNA, and the finally adopted primers are described in Table 1.

The method for MCMV miRNA examination was modified from previous reports [31,32]. Briefly, total RNA was extracted from mock-infected MEFs or MEFs at 6, 12, 24, 48, 72, and 96 h post-infection (hpi) using the mirVana™ PARIS™ Kit (Ambion, Austin, TX, USA) according to manufacturer’s instructions. The complementary DNA (cDNA) synthesis reaction was performed using the One Step miRNA cDNA Synthesis Kit (HAI-gene Bio Inc., Harbin, China), in which the poly(A) tailing of the miRNAs was followed by reverse transcription (RT) using a tagged poly(T) primer (5′-CAGGTCCAGTTTTTTTTTTTTTTTVN-3′). The generated cDNA was then used for miRNA primer screening in real-time quantitative polymerase chain reaction (qPCR) assays, which were performed in 20 μL with 1 μL prepared cDNA, 0.5 μL each primer, and 10 μL SYBR Green PCR Master Mix (TOYOBO, Osaka, Japan) under the following conditions: 95 °C for 1 min, 40 cycles of 95 °C for 15 s, 58 °C for 30 s, and 72 °C for 30 s in C1000™ Touch (Bio-Rad, Hercules, CA, USA). The relative expression level of each miRNA was normalized to that of U6 (forward primer, 5′-CGCTTCGGCAGCACATATACTA-3′, and reverse primer, 5′-CGCTTCACGAATTTGCGTGTCA-3′, range from 15 to 17 cycle threshold (CT) using the 2^−ΔΔCT^ method.

### 2.3. Examination of MCMV MiRNAs in Different Animal Models

All animal experiments were performed in strict accordance with Institutional Animal Care and Use Committee (IACUC) guidelines, and were approved by the Ethics Committee at the Beijing Institute of Transfusion Medicine, Beijing, China (IACUC of AMMS-13-2015-002).

BALB/c mice (male, 3–4 weeks) were obtained from Vital River (Beijing, China) and divided into two groups prior to infection (acute infection group and viral reactivation group). All mice were injected intraperitoneally with 10^5^ PFU of MCMV. For the acute infection group, mice were euthanized on day 5 post-infection; mice in the viral reactivation group were maintained for 25 weeks to establish latency as previously described [33,34]. MCMV recurrence in the viral reactivation group was induced as we previously described [28]: BALB/c recipient mice latently infected with MCMV underwent 700cGy total body irradiation and received an intravenous (IV) injection of 1 × 10^7^ bone marrow cells plus 1 × 10^7^ spleen cells from C57BL/6 mice (male, Vital River). The surviving recipients were euthanized using CO_2_ on day 28 post-transplantation.

The organs from each group were analyzed to profile MCMV miRNAs. Briefly, the livers, lungs, and blood were collected. Total RNA extraction and cDNA synthesis were performed as described above. Then, qPCR was performed to measure the expression of MCMV miRNAs based on our validated methods in vitro. The U6 expression level of each liver or lung was maintained at a similar level. A target miRNA was considered detectable if the CT value was less than 37 and a specific melt curve was observed.

### 2.4. Transfection and Quantification of Viral Loads for MiRNAs in Group 1

MEF cells were cultured in 24-well plates and transiently transfected with specific MCMV miRNA inhibitors (miR-m01-2-5p, miR-m01-2-3p, miR-m01-4-5p, miR-m21-1, miR-M23-2-5p, miR-M23-2-3p, miR-M44-1, miR-m88-1-3p, miR-M95-1-5p, miR-m108-2-5p.2, and miR-m108-2-3p), or a negative control RNA (NC-RNA) (all from RiboBio, Guangzhou, China) at a concentration of 150 nM for 12 h prior to infection using a transfection reagent (Micropoly-transfecter^TM^, Nantong, China). Each well containing cells was incubated with MCMV at a multiplicity of infection (MOI) of 0.5 for 12 h, and then the culture medium was replaced with fresh medium. After MCMV infection for 72 h, total DNA was isolated from the supernatants of infected MEFs using a DNA extraction kit (BioTeke Corporation, Beijing, China). Viral copies were measured by performing absolute qPCR with primers against the MCMV ie1 gene (forward primer, 5′-GTGGGCATGAAGTGTGGGTA-3′, and reverse primer, 5′-CGCATCGAAAGACAACGCAA-3′). In another set of transfections, cells transfected with either 150 nM inhibitor of two representative MCMV miRNAs (miR-m01-4-5p, miR-m21-1) or NC-RNA were infected with MCMV, and the titers were determined by TCID_50_ as described above.

### 2.5. Transfection and Quantification of Viral Loads for MiRNAs in Group 2

MEF cells were transfected with specific mimics of viral miRNAs (miR-m01-1, miR-m01-3-5p, miR-m01-3-3p, miR-m01-4-3p, miR-m22-1, miR-M23-1-5p, miR-M23-1-3p, miR-M55-1, miR-m59-1, miR-m59-2, miR-M87-1, miR-m88-1-5p, miR-M95-1-3p, miR-m107-1-5p, miR-m107-1-3p, miR-m108-1-5p, miR-m108-1-3p and miR-m108-2-5p.1) or NC-RNA (all from RiboBio) at a concentration of 100 nM. Cells were then infected with MCMV, and the viral copies were determined by qPCR in the same manner as described above.

To further investigate the effects of viral miRNAs on MCMV replication, MEFs were transfected with mimics, inhibitors of two representative MCMV miRNAs (miR-m01-3-5p and miR-M23-1-5p), or NC-RNA at two concentrations (30 and 100 nM). The titers were examined using TCID_50_ assays as described above.

### 2.6. Effects of Agomir Administration on MCMV Recurrence

**Figure 1:**
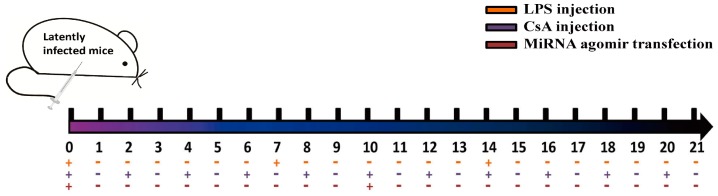
A schematic diagram depicting agomir transfection into mouse livers is shown. Briefly, BALB/c mice with latent murine cytomegalovirus (MCMV) infection were divided equally into three groups (eight per group). All mice were injected intraperitoneally with lipopolysaccharide (LPS; 15 μg/kg weekly) and cyclosporin A (CsA; 20 mg/kg/d every other day) for three weeks. To overexpress viral miRNAs, the mice received hydrodynamic injections of 2 mL phosphate-buffered saline (PBS) containing miR-m01-3-5p, miR-M23-1-5p, or negative control agomir (NC-agomir) on day 0 and day 10.

The induction of MCMV reactivation using lipopolysaccharide (LPS) and cyclosporin A (CsA) (both from Sigma-Aldrich, St. Louis, MO, USA) was adapted from previous reports [35,36]. Briefly, BALB/c mice with latent MCMV infection were divided equally into three groups. All mice were injected intraperitoneally with LPS (15 μg/kg, weekly) and CsA (20 mg/kg/d, every other day) for three weeks. To overexpress viral miRNAs, the mice received hydrodynamic injections of 2 mL phosphate buffered saline (PBS) containing 300 μg miR-m01-3-5p, miR-M23-1-5p, or negative control agomir (NC-agomir) on day 0 and day 10 (see Figure 1 for details). On day 21, all animals were euthanized using CO_2_, and the livers were collected to determine viral loads as described above. Livers from latently infected mice without drug administration were also measured as a negative control.

### 2.7. Immunohistochemistry

To visualize viral replication in animal livers, immunohistochemical staining targeting the MCMV-gB antigen was performed as described previously [28]. Briefly, formalin-fixed and paraffin-embedded liver sections were de-paraffinized and hydrated using graded alcohol washes. The samples were blocked with PBS containing with 0.2% Triton X-100 and 10% normal horse serum at room temperature for 1 h. Then, samples were incubated with primary antibody (rabbit anti-MCMV-Gb polyclonal antibody; custom antibody services provided by ABclonal Technology, Wuhan, China) overnight at 4 °C. After washing, all slides were incubated with the secondary antibody from a Dako kit (REAL™ EnVision™ Rabbit/Mouse, K5007, Copenhagen, Denmark), followed immediately by the application of 3,3′-diaminobenzidine and hematoxylin.

### 2.8. Statistical Analysis

All data are described as the mean ± standard deviation (SD). Statistical analyses were performed using SPSS V.17 (SPSS Inc., Chicago, IL, USA). Significant differences were calculated using one-way analysis of variance (ANOVA) followed by a Dunnett’s *t*-test or Kruskal–Wallis test followed by the Mann–Whitney *U* test. *p*-values < 0.05 were considered statistically significant.

## 3. Results

### 3.1. MCMV MiRNAExpression during Lytic Infection In Vitro

**Figure 2:**
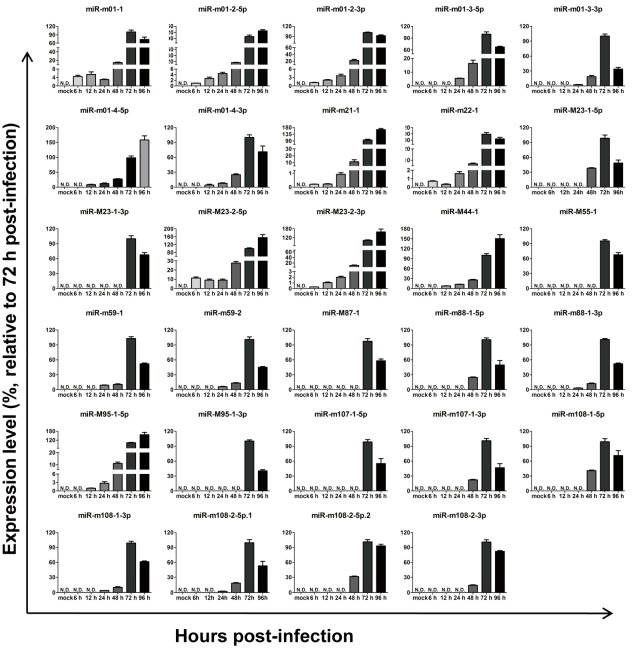
Expression kinetics of murine cytomegalovirus MCMV-encoded microRNAs (miRNAs) in vitro. Mouse embryonic fibroblasts (MEFs) were infected with MCMV, and miRNA profiles were determined using a revised real-time quantitative PCR (RT-qPCR) protocol at the indicated time points post-infection. MEFs with mock infection were used as negative controls. The results are shown as fold-change values relative to the expression levels at 72 h post-infection (hpi). Data presented are the result of three independent experiments and are shown as the mean ± standard deviation (SD). N.D.: not detected.

To investigate the expression kinetics of miRNAs encoded by MCMV in vitro, MEF cells were infected with MCMV (Smith strain), and total RNA was extracted at different time points following infection. Based on melt curve analyses, the levels of all29 MCMV miRNAs recorded in miRBase (Version 21) were examined using real-time quantitative polymerase chain reaction (RT-qPCR), as previously described [31,32]. After aggregating the data, the relative expression levels were calculated as fold changes relative to level at 72 hpi (Figure 2). The results showed that all 29 miRNAs were detectable at 72 and 96 hpi, although with different kinetics and expression onsets.

### 3.2. In Vivo MiRNA Expression Profiles Differ from In Vitro Profiles

We next tested the expression of MCMV miRNAs in vivo. Multiple organs or cell types are invaded during primary infection. However, only a few of these (e.g., liver, lung, and peripheral blood leukocytes) have been reported as sources of latent infection at high risk for viral recurrence [37,38,39]. Additionally, studies investigating rats infected with rat cytomegalovirus revealed viral miRNA expression profiles that were tissue-specific and associated with different states of viral infection [40]. Therefore, miRNA profiles were measured in the livers, lungs, and blood at both primary and recurrent infection.

**Figure 3:**
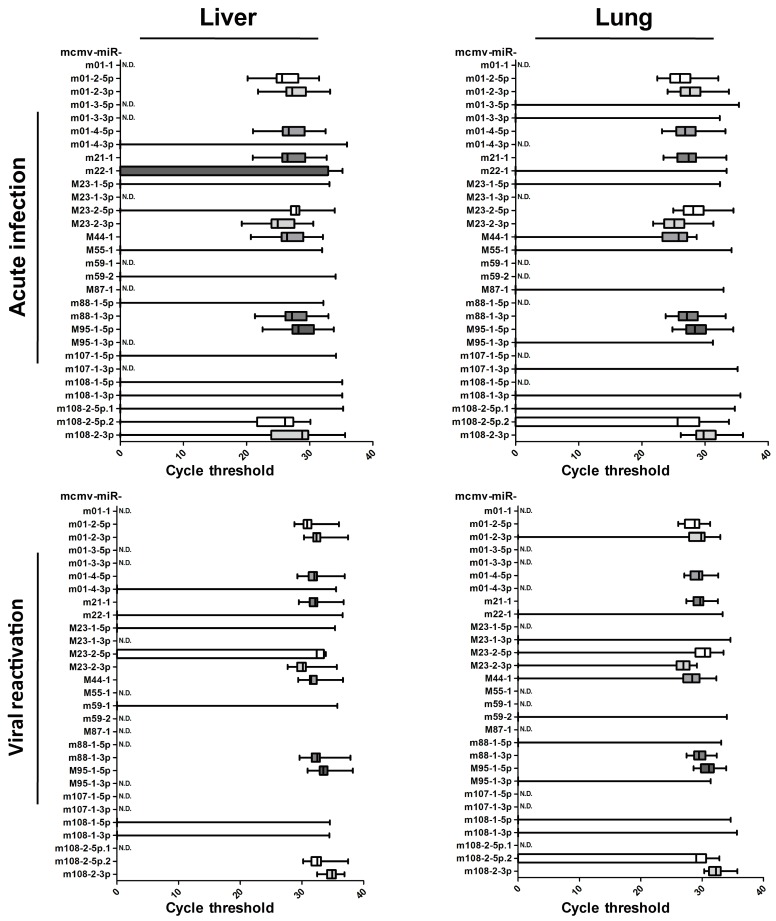
Examination of MCMV miRNA profiles in livers and lungs from two animal models. To test the expression of MCMV miRNAs in vivo, livers and lungs from acute infection/viral recurrence models were collected and performed to ascertain the miRNA profiles by RT-qPCR. The cycle threshold values are shown. The establishment of the two models was described in the Materials and Methods in detail.

Samples from acute infection or viral recurrence models were collected and prepared to ascertain the miRNA profiles, as described above (Table 2, and detailed in Table S1). The results showed that viral miRNA expression in the livers and lungs were readily detectable but were much more reduced in the blood. To clearly illustrate the MCMV miRNA profiles in vivo, the qPCR CT values are shown in Figure 3. These results revealed no obvious differences between liver and lung expression. Overall, the miRNA profiles were very similar during primary infection and viral recurrence, suggesting that the viral miRNAs utilize similar operational modes during these two lytic infection periods.

Although miRNA expression encoded by MCMV has been observed in infected cells, the results obtained from solid organs in both models indicated that the levels of over half of viral miRNAs were below the threshold of detection. Based on these results, the in vivo miRNA expression profiles were inconsistent with those detected in vitro. The selective expression of MCMV-encoded miRNAs implies that these miRNAs are involved in the complex regulation of viral activity during in vivo infection. Based on expression levels in the livers and lungs, MCMV miRNAs are classified into two groups. Group 1 MCMV miRNAs (11 miRNAs: miR-m01-2-5p, miR-m01-2-3p, miR-m01-4-5p, miR-m21-1, miR-M23-2-5p, miR-M23-2-3p, miR-M44-1, miR-m88-1-3p, miR-M95-1-5p, miR-m108-2-5p.2, and miR-m108-2-3p) were detected in more than 50% of animals, whereas Group 2 MCMV miRNAs (18 miRNAs: miR-m01-1, miR-m01-3-5p, miR-m01-3-3p, miR-m01-4-3p, miR-m22-1, miR-M23-1-5p, miR-M23-1-3p, miR-M55-1, miR-m59-1, miR-m59-2, miR-M87-1, miR-m88-1-5p, miR-M95-1-3p, miR-m107-1-5p, miR-m107-1-3p, miR-m108-1-5p, miR-m108-1-3p, and miR-m108-2-5p.1) were detected in less than 50% of animals. Considering that these two groups of miRNAs appear to be differentially regulated by MCMV during in vivo infection, it is possible that they play distinct roles during viral replication.

### 3.3. The Inhibition of Group 1 Viral MiRNAs Exerts Few Effectson MCMV Replication In Vitro

**Figure 4:**
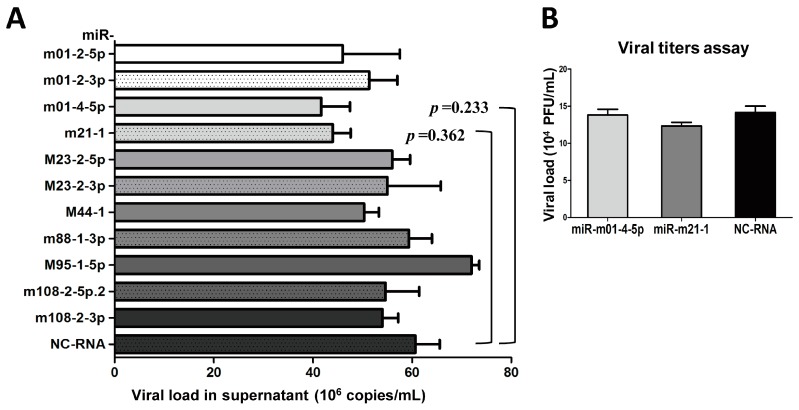
The inhibition of Group 1 viral miRNAs exerts minimal effects on viral production. (**A**) MEF cells were cultured in 24-well plates and transiently transfected with specific inhibitors of miRNAs from Group 1 or negative control RNA (NC-RNA). After 72 h of MCMV infection, viral copies were quantified by qPCR. (**B**) Two inhibitors of representative miRNAs (miR-m01-4-5p and miR-m21-1) were further examined using TCID_50_ (50% tissue culture infective dose) assays. The results are shown as the mean ± SD. The assays were performed in triplicate wells, and data were confirmed in three independent experiments. *p* < 0.05 for viral miRNA-transfected versus NC-RNA-treated MEFs. PFU: Plaque-forming unit.

As mentioned above, we found that MCMV achieves its replication cycle in vivo with the simultaneous expression of viral miRNAs in Group 1, whereas the expression of the miRNAs in Group 2 nearly disappeared. We speculated that these changes in miRNA expression were correlated with MCMV production in vivo. Thus, the next set of experiments was designed to test whether inhibition of Group 1 miRNAs impacts MCMV replication. For this purpose, 11 inhibitors targeting Group 1 MCMV miRNAs and NC-RNA were transfected into MEF cells, followed by MCMV infection. Viral copy numbers were examined in each sample by performing qPCR at three days post-infection. No significant attenuation was observed during MCMV propagation (*p* > 0.05, Figure 4A). The effects of two MCMV miRNAs that elicited a mild inhibition of MCMV production based on qPCR (miR-m01-4-5p and miR-m21-1) were further examined using titer assays, although the results showed no significant differences (*p* > 0.05, Figure 4B). These findings indicate that MCMV-encoded miRNAs in Group 1 likely do not play a large role in viral replication.

### 3.4. The Overexpression of Several Viral miRNAs in Group 2 Inhibits MCMV Production In Vitro

**Figure 5:**
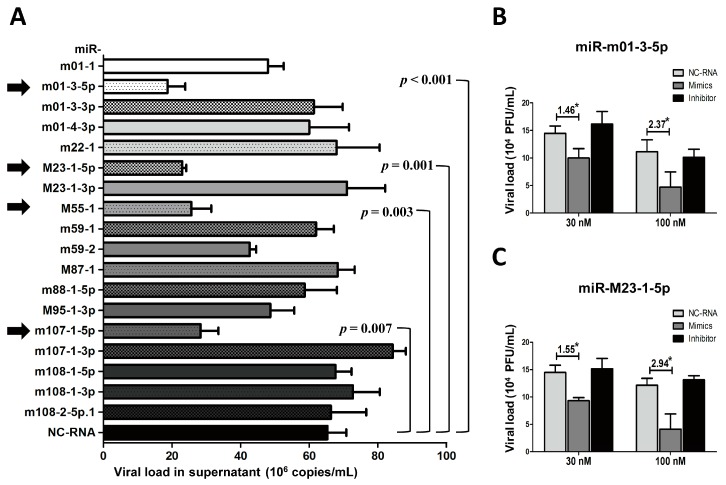
The overexpression of several Group 2 viral miRNAs inhibits MCMV replication. MEF cells were transfected with specific mimics of viral miRNAs or NC-RNA, followed by MCMV infection. Viral copies were quantified at 72 hpi using qPCR (**A**). To further investigate the effects of viral miRNAs on MCMV replication, the mimics, inhibitors of two representative miRNAs (miR-m01-3-5p and miR-M23-1-5p), or NC-RNA were transfected into MEFs at two concentrations (30 and 100 nM), followed by MCMV infection. Viral titers were examined using TCID_50_ assays, as described above (**B**,**C**). The results are shown as the mean ± SD. The assays were performed in triplicate wells, and all data were confirmed with three independent experiments. The different fold changes are shown above the horizontal bar. * *p* < 0.05 for viral miRNA-transfected versus NC-RNA-treated MEFs.

To test the effects of Group 2 miRNAs on viral replication, we performed a similar set of experiments used to test the Group 1 miRNAs. Group 2 miRNA mimics and NC-RNA were transfected into MEF cells, followed by MCMV infection. Viral copies were quantified at 72 hpi using qPCR (Figure 5A). In contrast with previous observations with Group 1, MEFs transfected with miR-m01-3-5p, miR-M23-1-5p, miR-M55-1, and miR-m107-1-5p showed statistically lower copy numbers of MCMV than MEFs transfected with NC-RNA (29%, 29%, 39%, and 43% versus control, respectively).

To further investigate the effects of viral miRNAs on MCMV replication, TCID_50_ assays were also performed on MCMV-infected cells transfected with the mimics, inhibitors of two representative miRNAs (miR-m01-3-5p and miR-M23-1-5p), or NC-RNA at two concentrations (30 and 100 nM) (Figure 5B,C). The results showed that overexpression of miR-m01-3-5p or miR-M23-1-5p inhibited viral replication in a dose-dependent manner.

### 3.5. MCMV MiRNA Agomirs Reduce MCMV Recurrence In Vivo

In the experiments presented above, the overexpression of several miRNAs in Group 2 suppressed viral replication in MEF cells—a phenomenon that could represent a novel approach for controlling MCMV infection. As miRNAs encoded by herpesvirus may be associated with the establishment and maintenance of latency, we examined the influence of miR-m01-3-5p and miR-M23-1-5p agomirs in an animal model of MCMV recurrence. Transfections into live mice were performed via hydrodynamic injection, as previously described [41,42].

**Figure 6:**
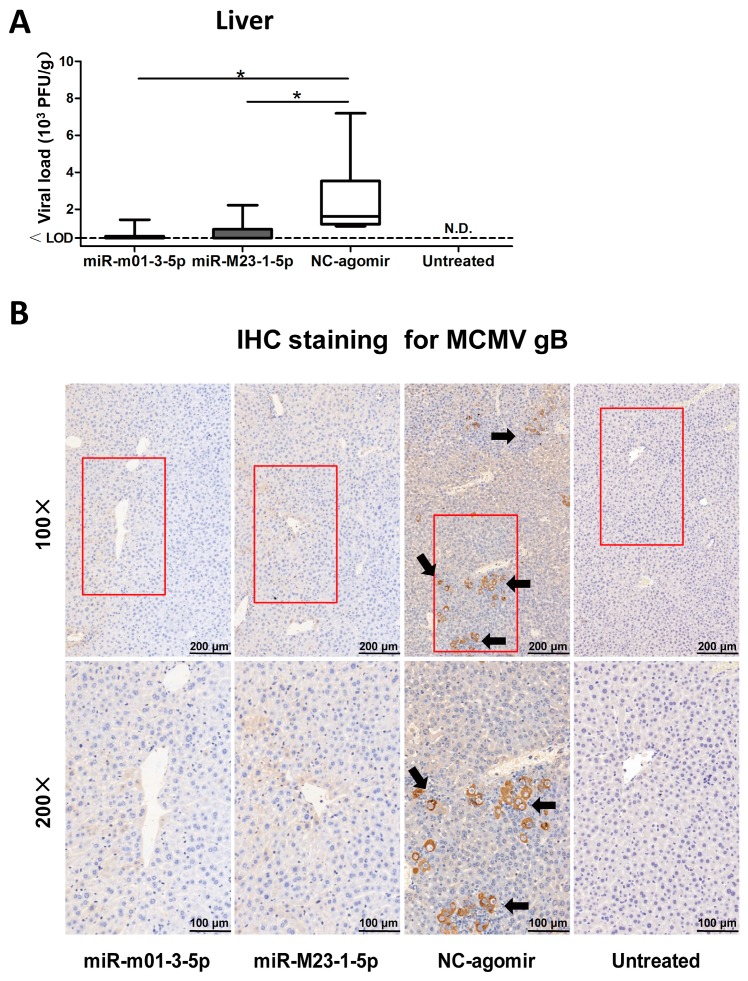
MCMV miRNA agomirs reduce MCMV recurrence in vivo. (**A**) After viral reactivation and transfection, livers were collected and analyzed to assess viral load; livers from latently-infected mice without drug administration were also measured as negative control. (**B**) To visualize viral reactivation in animal livers, immunohistochemical (IHC) staining targeting MCMV-gB antigen was performed (black arrow: recurrent virus; red box: the selected magnified area; magnification: 100×/200×). Data are based on two identical experiments. Dashed lines indicate the limit of detection (LOD) for the titer assay. * *p* < 0.05 for miRNA agomir-transfected group versus NC-agomir-transfected group.

Viral reactivation was induced in latently-infected BALB/c mice by injecting LPS and CsA. To manipulate viral miRNA expression in vivo, the mice received hydrodynamic injections of miR-m01-3-5p agomir, miR-M23-1-5p agomir, or NC-agomir (detailed in Figure 1). The livers were collected and analyzed to assess viral load, and livers from latently-infected mice without drug administration were also measured as a negative control (Figure 6A). The results showed that three groups of mice receiving drug administration exhibited viral reactivation. Importantly, mice in the two agomir-treated groups showed reduced recurrent MCMV titers compared with those in the NC-agomir-treated group.

To further demonstrate the efficacy of miR-m01-3-5p and miR-M23-1-5p agomirs as protective agents against viral invasion, immunohistochemical staining with anti-MCMV-gB polyclonal antibodies was performed to visualize virus production in the liver (Figure 6B). Mice in the NC-agomir group exhibited clearly higher viral loads in their livers, in accordance with the viral titer data. Therefore, based on our observations of miR-m01-3-5p and miR-M23-1-5p agomir function, these agomirs provide protection against MCMV reactivation in animals.

## 4. Discussion

Over the past decade, a large number of viral miRNAs and their potential binding sites have been predicted using a variety of approaches; however, the validity and functional relevance of the vast majority of these predictions remain poorly understood [10,43]. This is particularly true for in vivo HCMV infection, due to both the rigorous species specificity of HCMV and the complex interactions between viral and host factors [9]. In this study, we describe in vitro and in vivo MCMV miRNA expression profiles. Specifically, in vivo MCMV miRNA expression profiles were quite different from those detected in vitro. Furthermore, the overexpression of two viral miRNAs that are rarely detectable during in vivo infection reduced the efficiency of MCMV replication both in vitro and vivo. The findings presented here point to a novel antiviral strategy for defending against CMV infection.

The dynamic expression of MCMV-encoded miRNAs was first described during lytic infection in MEFs. All 29 miRNAs were detectable at 72 and 96 hpi, at which point the next generation of viruses is released, causing viral titers to rapidly increase. Based on two animal models, we characterize the expression of MCMV miRNAs in the context of both acute infection and viral reactivation. Specifically, we tested several organs (livers, lungs, and blood) to ascertain tissue specificity. Eighteen MCMV miRNAs were readily detectable in vitro, but were rarely detectable in livers/lungs with lytic viral replication. This is somewhat similar to studies investigating rat/rhesus cytomegalovirus, in which they observed the differential expression of several miRNAs in fibroblasts and salivary glands [40,44]. These differences in miRNA expression profiles may reflect distinct viral functional modes in different infection environments. We also found that miRNA profiles were highly similar during primary infection and viral recurrence—both periods in which a large number of infectious viruses are produced, suggesting viral miRNA expression profiles correlate with viral replication. Our results and previous studies highlight the importance of studying in vivo CMV miRNA profiles to identify the mechanism of selective expression. Notably, we also observed relatively lower unstable miRNA expression in the blood, which differs greatly from observations in lungs and livers. Several circulating HCMV miRNAs have been previously described as potential biomarkers, and were associated with hypertension, diabetes, and transplantation [25,45,46]. In those studies, miRNA expression was not detected in certain patient blood samples, consistent with our observations. Therefore, viral miRNA expression in the blood appears to change constantly and may be subject to multiple regulatory mechanisms [25]. 

We next investigated whether changes in viral miRNAs affect MCMV replication. Based on miRNA expression patterns, we classified MCMV miRNAs into two groups. Group 1 contained MCMV miRNAs that were detected in most animals with lytic infection, and Group 2 contained those that are rarely detected. We then proposed a hypothesis to explain our observations of viral miRNAs in vivo—namely, that miRNAs in Group 1 are positively associated with MCMV production and miRNAs in Group 2 negatively influence viral replication. In our transfection experiments for Group 1, two inhibitors targeting miR-m01-4-5p and miR-m21-1 showed very mild antiviral effects based on qPCR, and no statistically significant differences were observed for the qPCR or titer assays. In previous investigations testing the biological effects of MCMV miRNAs, miR-m01-4-5p (documented as miR-m01-4) accounted for as much as 40% of all viral miRNAs during in vitro lytic infection [26]. Similar to our findings, miR-m01-4-5p as well as other miRNAs in Group 1 also exhibited little impact on viral load. Therefore, although the miRNAs in Group 1 were strongly expressed during in vivo infection, they do not appear to play a large role in the regulation of viral replication. In a recent clinical study, miR-UL22A-5p—which is encoded by HCMV—was used as a biomarker during transplantation [25]. The authors demonstrated a significant regulatory role for miR-UL22A-5p in host gene expression by targeting the transcription factor C-MYC as well as many proteins involved in antigen presentation. Therefore, the roles of CMV-encoded miRNAs are not limited to the regulation of viral replication, and they may benefit viral invasion in other ways, such as immune modulation and immune evasion.

We next investigated the effects of the Group 2 miRNAs on MCMV propagation. In contrast with the results obtained with Group 1, the overexpression of four miRNAs in Group 2 resulted in statistically significant reductions in the viral copies in infected MEFs. The antiviral effects of two miRNAs were further confirmed using TCID_50_ assays. It is generally known that HCMV is never cleared following primary infection, but rather establishes latency and persists for the lifetime of the host [9]. To maintain latency, multiple strategies are employed to continuously repress viral gene expression and prevent the virus from restarting the lytic cycle [40]. Therefore, it is reasonable to propose that viral miRNAs are involved in establishing latency based on their ability to inhibit viral replication, exactly in line with the principle that diminished viral activity benefits survival in the face of a hostile host immune system [47]. In previous herpesvirus studies, many viral miRNAs were found to suppress viral production by targeting the expression of viral transactivators themselves, including latency-associated transcript miRNA (miR-LAT) which is encoded by herpes simplex virus type 1 (HSV-1) and targets infected cell protein 0 (ICP0); miR-K10-6-3p, which is encoded by Kaposi’s sarcoma herpesvirus (KSHV) and targets Rta and Zta; miR-BHRF-1 and miR-BART15, which are encoded by Epstein–Barr virus (EBV) and target BZLF1 and BRLF1, respectively [16]. For HCMV, miR-UL112, miR-US25-1, and miR-US25-2 were reported to repress viral DNA synthesis and contribute to latency [13,14,17,19]. In addition, HCMV, KSHV, and EBV can also reduce virus-infected cell death by encoding miRNAs that target the natural killer (NK) cell-activating ligand MICB [24]. Overall, the selective expression of viral miRNAs may represent a non-immunogenic and convenient strategy to defend immune surveillance and stably alter the cellular environment during latency, which is a common feature of most herpesviruses [40,48].

Rather than focusing on viral miRNAs that are expressed during active infection in vivo, we chose to investigate the role of miRNAs that were undetectable in most of animals, as a way to identify potential miRNA-based-therapies to reduce HCMV infection severity. In a recent study, in vivo expression of HCMV miRNAs during latency and reactivation were evaluated, highlighting their important roles in the regulation of lytic/latent phase [49]. Due to deficient animal models for HCMV infection, as well as the unawareness of a mechanism for CMV miRNA expression, further investigation using MCMV-infected mice is still needed to gain a clear understanding of miRNA expression profiles and functional relevance in viral replication, latency, and pathogenesis. 

Importantly, we demonstrate that the overexpression of two viral miRNAs using agomir transfection can control acute MCMV infection in animals. Antiviral drugs currently used for the treatment of CMV infection inhibit viral replication by interfering with viral DNA polymerase [50]. Although they have proven effective, drug administration still has serious limitations in clinical practice due to significant adverse effects. For ganciclovir, 7–35% of recipients receiving transplantation and ganciclovir administration develop leukopenia [51,52,53], and 3–15% experience neutropenia [52,54,55]. In addition, an increasing number of drug-resistant strains have been isolated, which are primarily due to mutations in the *UL97* and *UL54* genes [56,57]. Therefore, it is necessary to develop new approaches for controlling and preventing CMV infection. To date, therapeutic miRNAs have emerged as novel and promising targets for antiviral strategies. In previous studies, miR-122 (a liver-specific miRNA) was shown to regulate HCV replication [58]. Treatment with an miR-122 antagonist resulted in excellent improvement in HCV-infected chimpanzees and humans [59]. However, interventions targeting miRNAs encoded by the host may result in side effects, suggesting that the manipulation of viral miRNAs may have more treatment potential. Additionally, the overexpression of MCMV miRNAs reduced viral load less dramatically in vivo than in vitro, suggesting the need for new methods of drug administration. Recent studies employing lipid nanoparticle-encapsulated small interfering RNAs (siRNAs) targeting the Makona outbreak strain of Ebola virus (EBOV) represent the first successful demonstration of therapeutic anti-EBOV therapies [60]. This study highlights the rapid development of lipid nanoparticle-delivered siRNAs for the treatment of viral infections.

## 5. Conclusions

In conclusion, our study explores MCMV miRNAs profiles in vitro and vivo. Importantly, the overexpression of several viral miRNAs reduced viral replication, representing a novel antiviral strategy involving miRNA-based treatment. Further research to characterize the role of CMV-encoded miRNAs in viral invasion is required, and may contribute to the development of new therapeutics that target and exploit this interesting relationship.

## Figures and Tables

**Table 1:** Primers used to examine murine cytomegalovirus (MCMV)-encoded microRNAs (miRNAs) in real-time quantitative polymerase chain reaction (qPCR) assays.

MiRNA Name	Accession Number	Melting Temperature (°C)	Primer F (5′–3′)	Primer R (5′–3′)	Group
miR-m01-1	MIMAT0005533	71.5	GCAGAGAGGAGAATAACGTC	GTCCAGTTTTTTTTTTTTTTTCCGT	2
miR-m01-2-5p	MIMAT0005534	72	CAGGAAGAGAATCGGGTTG	GTCCAGTTTTTTTTTTTTTTTACCGT	1
miR-m01-2-3p	MIMAT0005535	72.5	GCGTTCGACACGGTTTCC	GTCCAGTTTTTTTTTTTTTTTCGAAG	1
miR-m01-3-5p	MIMAT0005536	74	AGCGGTGAAGCGACTGTTGC	GTCCAGTTTTTTTTTTTTTTTCGAG	2
miR-m01-3-3p	MIMAT0005537	75	AGCGAGGAACGCTCGCTTCAC	GGTCCAGTTTTTTTTTTTTTTTGCC	2
miR-m01-4-5p	MIMAT0005538	75	CGCAGTCCTATGCTAACAC	GGTCCAGTTTTTTTTTTTTTTTCAC	1
miR-m01-4-3p	MIMAT0005539	74	AGCGCCGCGTGGTAGCAT	GGTCCAGTTTTTTTTTTTTTTTGTTCT	2
miR-m21-1	MIMAT0005540	72.5	GCAGATAGGGGACACGTTC	CAGTTTTTTTTTTTTTTTCGGCTTG	1
miR-m22-1	MIMAT0005541	73.5	CAGTTCCCGTCCGTACCGA	CCAGTTTTTTTTTTTTTTTGGCCT	2
miR-M23-1-5p	MIMAT0005542	73	ACTCGGTACGGACGGGGAA	GTCCAGTTTTTTTTTTTTTTTACGGT	2
miR-M23-1-3p	MIMAT0005543	75	CTCCTGCGTCGGCCCGAG	GTCCAGTTTTTTTTTTTTTTTGGC	2
miR-M23-2-5p	MIMAT0005544	73	CAGTGAACGTGTCCCCTATC	GGTCCAGTTTTTTTTTTTTTTTCCA	1
miR-M23-2-3p	MIMAT0005545	74.5	AGCAGATGGGGGCCTCGGT	AGTTTTTTTTTTTTTTTCCGCTTGA	1
miR-M44-1	MIMAT0005546	74	CGCAGTATCTTTTTCCAGAG	AGGTCCAGTTTTTTTTTTTTTTTACC	1
miR-M55-1	MIMAT0005547	75.5	GGTGATCGGCGTGCTA	GTCCAGTTTTTTTTTTTTTTTACGG	2
miR-m59-1	MIMAT0005548	73.5	TTAGCAGTGCCTCGACCGT	GGTCCAGTTTTTTTTTTTTTTTCTGA	2
miR-m59-2	MIMAT0005549	73.5	GCCCGAAGAGCCCTC	AGTTTTTTTTTTTTTTTGGCTCTGT	2
miR-M87-1	MIMAT0005550	74.5	CCGTCGGCAGCG	GGTCCAGTTTTTTTTTTTTTTTGCT	2
miR-m88-1-5p	MIMAT0005551	73	CAGATGACCGACCCCCTGA	CCAGTTTTTTTTTTTTTTTCCGATGT	2
miR-m88-1-3p	MIMAT0005552	74	AGCAGCAGAAGTCGATGT	GTCCAGTTTTTTTTTTTTTTTAGACC	1
miR-M95-1-5p	MIMAT0005553	73.5	AGGGTCGTGGGCTTGTGT	CAGTTTTTTTTTTTTTTTCAAGCGA	1
miR-M95-1-3p	MIMAT0005554	75	AGCGACGTCGGACCGCGA	CCAGTTTTTTTTTTTTTTTGCCGT	2
miR-m107-1-5p	MIMAT0005555	75.5	CGGTCACTCGTCTCGA	CCAGTTTTTTTTTTTTTTTGGTGACT	2
miR-m107-1-3p	MIMAT0005556	75.5	AGTGCTCGCGTCGAGTGACC	GGTCCAGTTTTTTTTTTTTTTTGAG	2
miR-m108-1-5p	MIMAT0005557	73	CAGTCACGAGCAACCGCCC	TCCAGTTTTTTTTTTTTTTTCATTTC	2
miR-m108-1-3p	MIMAT0005558	74	CAGTTTCTGACGGTGGCT	GTCCAGTTTTTTTTTTTTTTTCGAC	2
miR-m108-2-5p.1	MIMAT0005560	75	GGCGGTCACTCGAC	AGTTTTTTTTTTTTTTTCGGTGCT	2
miR-m108-2-5p.2	MIMAT0005559	74	TCACTCGTCGCGAGCGGT	GGTCCAGTTTTTTTTTTTTTTTGTGA	1
miR-m108-2-3p	MIMAT0005561	73.5	GTGACTCGAGACGAGTGA	GGTCCAGTTTTTTTTTTTTTTTACCG	1

**Table 2:** Viral miRNA profiles in the organs of mice suffering from acute infection or viral recurrence.

	Acute Infection Group (*N* = 15), *n* (%)			Viral Reactivation Group (*N* = 17), *n* (%)		
	Livers	Lungs	Blood	Livers	Lungs	Blood
miR-m01-1	0 (0)	0 (0)	0 (0)	0 (0)	0 (0)	0 (0)
miR-m01-2-5p	15 (100)	15 (100)	15 (100)	17 (100)	17 (100)	15 (88)
miR-m01-2-3p	15 (100)	15 (100)	3 (20)	17 (100)	14 (82)	1 (6)
miR-m01-3-5p	0 (0)	3 (20)	1 (7)	0 (0)	0 (0)	0 (0)
miR-m01-3-3p	0 (0)	1 (7)	0 (0)	0 (0)	0 (0)	1 (6)
miR-m01-4-5p	15 (100)	15 (100)	9 (60)	17 (100)	17 (100)	7 (41)
miR-m01-4-3p	3 (20)	0 (0)	4 (27)	3 (18)	0 (0)	3 (18)
miR-m21-1	15 (100)	15 (100)	6 (40)	17 (100)	17 (100)	9 (53)
miR-m22-1	6 (40)	3 (20)	3 (20)	3 (18)	3 (18)	2 (12)
miR-M23-1-5p	2 (13)	1 (7)	0 (0)	3 (18)	0 (0)	1 (6)
miR-M23-1-3p	0 (0)	0 (0)	1 (7)	0 (0)	2 (12)	0 (0)
miR-M23-2-5p	14 (93)	15 (100)	8 (53)	12 (71)	14 (82)	9 (53)
miR-M23-2-3p	15 (100)	15 (100)	8 (53)	17 (100)	14 (82)	13 (76)
miR-M44-1	15 (100)	12 (80)	2 (13)	17 (100)	14 (82)	4 (24)
miR-M55-1	1 (7)	1 (7)	0 (0)	0 (0)	0 (0)	2 (12)
miR-m59-1	0 (0)	0 (0)	1 (7)	2 (12)	0 (0)	0 (0)
miR-m59-2	3 (20)	0 (0)	0 (0)	0 (0)	3 (18)	1 (6)
miR-M87-1	0 (0)	2 (13)	1 (7)	0 (0)	0 (0)	0 (0)
miR-m88-1-5p	1 (7)	0 (0)	0 (0)	0 (0)	3 (18)	0 (0)
miR-m88-1-3p	15 (100)	15 (100)	8 (53)	17 (100)	17 (100)	12 (71)
miR-M95-1-5p	15 (100)	15 (100)	3 (20)	17 (100)	17 (100)	7 (41)
miR-M95-1-3p	0 (0)	2 (13)	0 (0)	0 (0)	2 (12)	2 (12)
miR-m107-1-5p	3 (20)	0 (0)	1 (7)	0 (0)	0 (0)	1 (6)
miR-m107-1-3p	0 (0)	1 (7)	3 (20)	0 (0)	0 (0)	0 (0)
miR-m108-1-5p	3 (20)	0 (0)	0 (0)	2 (12)	2 (12)	0 (0)
miR-m108-1-3p	3 (20)	2 (13)	4 (27)	1 (6)	1 (6)	1 (6)
miR-m108-2-5p.1	2 (13)	3 (20)	1 (7)	0 (0)	0 (0)	0 (0)
miR-m108-2-5p.2	12 (80)	9 (60)	10 (67)	17 (100)	12 (71)	11 (65)
miR-m108-2-3p	12 (80)	15 (100)	7 (47)	17 (100)	17 (100)	15 (88)

*N*: Number of mice for each group; *n* (%): Number of mice with certain miRNA detected (*n*/*N* × 100%).

## Supplementary Materials

The following are available online at www.mdpi.com/1999-4915/9/5/118/s1, Table S1: Amplification targets.

## References

[1] Bate S.L., Dollard S.C., Cannon M.J. (2010). Cytomegalovirus seroprevalence in the United States: The national health and nutrition examination surveys, 1988–2004. Clin. Infect. Dis..

[2] Jackson S.E., Mason G.M., Wills M.R. (2011). Human cytomegalovirus immunity and immune evasion. Virus Res..

[3] Goodrich J.M., Bowden R.A., Fisher L., Keller C., Schoch G., Meyers J.D. (1993). Ganciclovir prophylaxis to prevent cytomegalovirus disease after allogeneic marrow transplant. Ann. Intern. Med..

[4] Reusser P., Einsele H., Lee J., Volin L., Rovira M., Engelhard D., Finke J., Cordonnier C., Link H., Ljungman P. (2002). Randomized multicenter trial of foscarnet versus ganciclovir for preemptive therapy of cytomegalovirus infection after allogeneic stem cell transplantation. Blood.

[5] Valdez O., Gaspar A., Dickson J., Weigert A., Machado D. (2003). Cytomegalovirus infection resistant to ganciclovir in a renal transplant patient. Transplant. Proc..

[6] Young P.G., Rubin J., Angarone M., Flaherty J., Penugonda S., Stosor V., Ison M.G. (2016). Ganciclovir-resistant cytomegalovirus infection in solid organ transplant recipients: A single-center retrospective cohort study. Transpl. Infect. Dis..

[7] Isada C.M., Yen-Lieberman B., Lurain N.S., Schilz R., Kohn D., Longworth D.L., Taege A.J., Mossad S.B., Maurer J., Flechner S.M. (2002). Clinical characteristics of 13 solid organ transplant recipients with ganciclovir-resistant cytomegalovirus infection. Transpl. Infect. Dis..

[8] Shen Z.Z., Pan X., Miao L.F., Ye H.Q., Chavanas S., Davrinche C., McVoy M., Luo M.H. (2014). Comprehensive analysis of human cytomegalovirus microRNA expression during lytic and quiescent infection. PLoS ONE.

[9] Ng K.R., Li J.Y., Gleadle J.M. (2015). Human cytomegalovirus encoded microRNAs: Hitting targets. Expert Rev. Anti-Infect. Ther..

[10] Hook L., Hancock M., Landais I., Grabski R., Britt W., Nelson J.A. (2014). Cytomegalovirus microRNAs. Curr. Opin. Virol..

[11] Dhuruvasan K., Sivasubramanian G., Pellett P.E. (2011). Roles of host and viral microRNAs in human cytomegalovirus biology. Virus Res..

[12] Huang Y., Qi Y., Ma Y., He R., Ji Y., Sun Z., Ruan Q. (2013). Down-regulation of human cytomegalovirus UL138, a novel latency-associated determinant, by hcmv-miR-UL36. J. Biosci..

[13] Stern-Ginossar N., Saleh N., Goldberg M.D., Prichard M., Wolf D.G., Mandelboim O. (2009). Analysis of human cytomegalovirus-encoded microRNA activity during infection. J. Virol..

[14] Grey F., Meyers H., White E.A., Spector D.H., Nelson J. (2007). A human cytomegalovirus-encoded microRNA regulates expression of multiple viral genes involved in replication. PLoS Pathog..

[15] Pavelin J., Reynolds N., Chiweshe S., Wu G., Tiribassi R., Grey F. (2013). Systematic microRNA analysis identifies ATP6V0C as an essential host factor for human cytomegalovirus replication. PLoS Pathog..

[16] Murphy E., Vanicek J., Robins H., Shenk T., Levine A.J. (2008). Suppression of immediate-early viral gene expression by herpesvirus-coded microRNAs: Implications for latency. Proc. Natl. Acad. Sci. USA.

[17] Jiang S., Qi Y., He R., Huang Y., Liu Z., Ma Y., Guo X., Shao Y., Sun Z., Ruan Q. (2015). Human cytomegalovirus microRNA miR-US25-1-5p inhibits viral replication by targeting multiple cellular genes during infection. Gene.

[18] Guo X., Qi Y., Huang Y., Liu Z., Ma Y., Shao Y., Jiang S., Sun Z., Ruan Q. (2015). Human cytomegalovirus miR-US33-5p inhibits viral DNA synthesis and viral replication by down-regulating expression of the host Syntaxin3. FEBS Lett..

[19] Qi M., Qi Y., Ma Y., He R., Ji Y., Sun Z., Ruan Q. (2013). Over-expression of human cytomegalovirus miR-US25-2-3p downregulates eIF4A1 and inhibits HCMV replication. FEBS Lett..

[20] Han S.J., Marshall V., Barsov E., Quinones O., Ray A., Labo N., Trivett M., Ott D., Renne R., Whitby D. (2013). Kaposi’s sarcoma-associated herpesvirus microRNA single-nucleotide polymorphisms identified in clinical samples can affect microRNA processing, level of expression, and silencing activity. J. Virol..

[21] Kim Y., Lee S., Kim S., Kim D., Ahn J.H., Ahn K. (2012). Human cytomegalovirus clinical strain-specific microRNA miR-UL148D targets the human chemokine RANTES during infection. PLoS Pathog..

[22] Kim S., Lee S., Shin J., Kim Y., Evnouchidou I., Kim D., Kim Y.K., Kim Y.E., Ahn J.H., Riddell S.R. (2011). Human cytomegalovirus microRNA miR-US4-1 inhibits CD8^+^ T cell responses by targeting the aminopeptidase ERAP1. Nat. Immunol..

[23] Nachmani D., Lankry D., Wolf D.G., Mandelboim O. (2010). The human cytomegalovirus microRNA miR-UL112 acts synergistically with a cellular microRNA to escape immune elimination. Nat. Immunol..

[24] Stern-Ginossar N., Elefant N., Zimmermann A., Wolf D.G., Saleh N., Biton M., Horwitz E., Prokocimer Z., Prichard M., Hahn G. (2007). Host immune system gene targeting by a viral miRNA. Science.

[25] Lisboa L.F., Egli A., O’Shea D., Asberg A., Hartmann A., Rollag H., Pang X.L., Tyrrell D.L., Kumar D., Humar A. (2015). Hcmv-miR-UL22A-5p: A biomarker in transplantation with broad impact on host gene expression and potential immunological implications. Am. J. Transplant..

[26] Dolken L., Perot J., Cognat V., Alioua A., John M., Soutschek J., Ruzsics Z., Koszinowski U., Voinnet O., Pfeffer S. (2007). Mouse cytomegalovirus microRNAs dominate the cellular small RNA profile during lytic infection and show features of posttranscriptional regulation. J. Virol..

[27] Gutermann A., Bubeck A., Wagner M., Reusch U., Menard C., Koszinowski U.H. (2002). Strategies for the identification and analysis of viral immune-evasive genes–Cytomegalovirus as an example. Curr. Top. Microbiol. Immunol..

[28] Deng J., Xiao J., Lv L., Ma P., Song X., Gao B., Gong F., Zhang Y., Xu J. (2016). Immunosuppressive therapy alleviates murine cytomegalovirus recurrence by reducing TNF-α post cell transplantation with lethal GVHD. Antiviral Res..

[29] Reed L.J., Muench H. (1938). A simple method of estimating fifty percent endpoints. Am. J. Hyg..

[30] Busk P.K. (2014). A tool for design of primers for microRNA-specific quantitative RT-qPCR. BMC Bioinform..

[31] Balcells I., Cirera S., Busk P.K. (2011). Specific and sensitive quantitative RT-PCR of miRNAs with DNA primers. BMC Biotechnol..

[32] Reichenstein I., Aizenberg N., Goshen M., Bentwich Z., Avni Y.S. (2010). A novel qPCR assay for viral encoded microRNAs. J. Virol. Methods.

[33] Palaniyandi S., Radhakrishnan S.V., Karlsson F.J., Stokes K.Y., Kittan N., Huber E., Hildebrandt G.C. (2013). Murine cytomegalovirus immediate-early 1 gene expression correlates with increased GVHD after allogeneic hematopoietic cell transplantation in recipients reactivating from latent infection. PLoS ONE.

[34] Gosselin J., Borgeat P., Flamand L. (2005). Leukotriene B4 protects latently infected mice against murine cytomegalovirus reactivation following allogeneic transplantation. J. Immunol..

[35] Ni D., Yu H., Zhang W., Gan L., Zhao J., Wang M., Chen J. (2013). A mouse model of interstitial pneumonitis induced by murine cytomegalovirus infection after allogeneic skin transplantation. Biomed. Res. Int..

[36] Cook C.H., Trgovcich J., Zimmerman P.D., Zhang Y., Sedmak D.D. (2006). Lipopolysaccharide, tumor necrosis factor α, or interleukin-1β triggers reactivation of latent cytomegalovirus in immunocompetent mice. J. Virol..

[37] Seckert C.K., Renzaho A., Tervo H.M., Krause C., Deegen P., Kühnapfel B., Reddehase M.J., Grzimek N.K. (2009). Liver sinusoidal endothelial cells are a site of murine cytomegalovirus latency and reactivation. J. Virol..

[38] Slobedman B., Cao J.Z., Avdic S., Webster B., McAllery S., Cheung A.K., Tan J.C., Abendroth A. (2010). Human cytomegalovirus latent infection and associated viral gene expression. Future Microbiol..

[39] Reddehase M.J., Podlech J., Grzimek N.K. (2002). Mouse models of cytomegalovirus latency: Overview. J. Clin. Virol..

[40] Meyer C., Grey F., Kreklywich C.N., Andoh T.F., Tirabassi R.S., Orloff S.L., Streblow D.N. (2011). Cytomegalovirus microRNA expression is tissue specific and is associated with persistence. J. Virol..

[41] Giladi H., Ketzinel-Gilad M., Rivkin L., Felig Y., Nussbaum O., Galun E. (2003). Small interfering RNA inhibits hepatitis B virus replication in mice. Mol. Ther..

[42] Zender L., Hutker S., Liedtke C., Tillmann H.L., Zender S., Mundt B., Waltemathe M., Gosling T., Flemming P., Malek N.P. (2003). Caspase 8 small interfering RNA prevents acute liver failure in mice. Proc. Natl. Acad. Sci. USA.

[43] Grey F., Nelson J. (2008). Identification and function of human cytomegalovirus microRNAs. J. Clin. Virol..

[44] Hancock M.H., Tirabassi R.S., Nelson J.A. (2012). Rhesus cytomegalovirus encodes seventeen microRNAs that are differentially expressed in vitro and in vivo. Virology.

[45] Mohammad A.A., Rahbar A., Lui W.O., Davoudi B., Catrina A., Stragliotto G., Mellbin L., Hamsten A., Rydén L., Yaiw K.C. (2014). Detection of circulating hcmv-miR-UL112-3p in patients with glioblastoma, rheumatoid arthritis, diabetes mellitus and healthy controls. PLoS ONE.

[46] Li S., Zhu J., Zhang W., Chen Y., Zhang K., Popescu L.M., Ma X., Lau W.B., Rong R., Yu X. (2011). Signature microRNA expression profile of essential hypertension and its novel link to human cytomegalovirus infection. Circulation.

[47] Poole E., Sinclair J. (2015). Sleepless latency of human cytomegalovirus. Med. Microbiol. Immunol..

[48] Piedade D., Azevedo-Pereira J.M. (2016). The Role of microRNAs in the Pathogenesis of Herpesvirus Infection. Viruses.

[49] Meshesha M.K., Bentwich Z., Solomon S.A., Avni Y.S. (2016). In vivo expression of human cytomegalovirus (HCMV) microRNAs during latency. Gene.

[50] Bowman L.J., Melaragno J.I., Brennan D.C. (2017). Letermovir for the management of cytomegalovirus infection. Expert Opin. Investig. Drugs.

[51] Asberg A., Humar A., Rollag H., Jardine A.G., Mouas H., Pescovitz M.D., Sgarabotto D., Tuncer M., Noronha I.L., Hartmann A. (2007). Oral valganciclovir is noninferior to intravenous ganciclovir for the treatment of cytomegalovirus disease in solid organ transplant recipients. Am. J. Transplant..

[52] Paya C., Humar A., Dominguez E., Washburn K., Blumberg E., Alexander B., Freeman R., Heaton N., Pescovitz M.D. (2004). Efficacy and safety of valganciclovir vs. oral ganciclovir for prevention of cytomegalovirus disease in solid organ transplant recipients. Am. J. Transplant..

[53] Winston D.J., Busuttil R.W. (2003). Randomized controlled trial of oral ganciclovir versus oral acyclovir after induction with intravenous ganciclovir for long-term prophylaxis of cytomegalovirus disease in cytomegalovirus-seropositive liver transplant recipients. Transplantation.

[54] Hodson E.M., Jones C.A., Webster A.C., Strippoli G.F., Barclay P.G., Kable K., Vimalachandra D., Craig J.C. (2005). Antiviral medications to prevent cytomegalovirus disease and early death in recipients of solid-organ transplants: A systematic review of randomised controlled trials. Lancet.

[55] Gane E., Saliba F., Valdecasas G.J., O’Grady J., Pescovitz M.D., Lyman S., Robinson C.A. (1997). Randomised trial of efficacy and safety of oral ganciclovir in the prevention of cytomegalovirus disease in liver-transplant recipients. The Oral Ganciclovir International Transplantation Study Group [corrected]. Lancet.

[56] Gilbert C., Boivin G. (2005). Human cytomegalovirus resistance to antiviral drugs. Antimicrob. Agents Chemother..

[57] Sullivan V., Biron K.K., Talarico C., Stanat S.C., Davis M., Pozzi L.M., Coen D.M. (1993). A point mutation in the human cytomegalovirus DNA polymerase gene confers resistance to ganciclovir and phosphonylmethoxyalkyl derivatives. Antimicrob. Agents Chemother..

[58] Jopling C.L., Yi M., Lancaster A.M., Lemon S.M., Sarnow P. (2005). Modulation of hepatitis C virus RNA abundance by a liver-specific microRNA. Science.

[59] Thibault P.A., Wilson J.A. (2013). Targeting miRNAs to treat hepatitis C virus infections and liver pathology: Inhibiting the virus and altering the host. Pharmacol. Res..

[60] Thi E.P., Mire C.E., Lee A.C., Geisbert J.B., Zhou J.Z., Agans K.N., Snead N.M., Deer D.J., Barnard T.R., Fenton K.A. (2015). Lipid nanoparticle siRNA treatment of Ebola-virus-Makona-infected nonhuman primates. Nature.

